# Supplementary material for: Quantification of Arbutin in Cosmetics, Drugs and Food Supplements by Hydrophilic-Interaction Chromatography
Source: Molecules. 2022 Sep 2;27(17):5673. doi: 10.3390/molecules27175673 (PMC9457821; doi:10.3390/molecules27175673)
Supplement: Supplementary file 1 [file molecules-27-05673-s001.zip › Supplementary File S10 - AGREE Report.pdf]

Analytical Greenness report sheet

05/07/2022 11:01:05

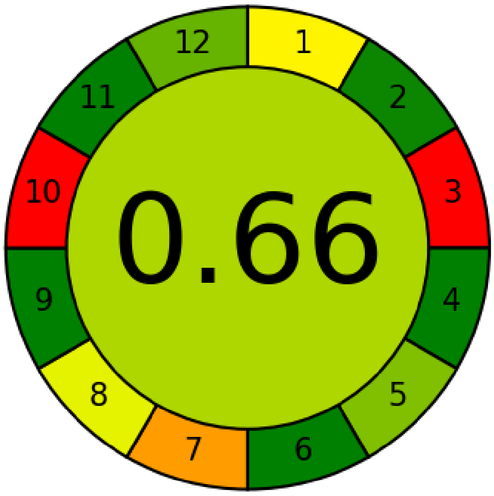

Criteria	Score	Weight
1. Direct analytical techniques should be applied to avoid sample treatment.	0.48	2
2. Minimal sample size and minimal number of samples are goals.	0.98	2
3. If possible, measurements should be performed in situ.	0.0	2
4. Integration of analytical processes and operations saves energy and reduces the use of reagents.	1.0	2
5. Automated and miniaturized methods should be selected.	0.75	2
6. Derivatization should be avoided.	1.0	2
7. Generation of a large volume of analytical waste should be avoided, and proper management of analytical waste should be provided.	0.31	2
8. Multi-analyte or multi-parameter methods are preferred versus methods using one analyte at a time.	0.55	2
9. The use of energy should be minimized.	1.0	2
10. Reagents obtained from renewable sources should be preferred.	0.0	2
11. Toxic reagents should be eliminated or replaced.	1.0	2
12. Operator's safety should be increased.	0.8	2
